# SWITCH: rationale, design, and implementation of a community, school, and family-based intervention to modify behaviors related to childhood obesity

**DOI:** 10.1186/1471-2458-8-223

**Published:** 2008-06-29

**Authors:** Joey C Eisenmann, Douglas A Gentile, Gregory J Welk, Randi Callahan, Sarah Strickland, Monica Walsh, David A Walsh

**Affiliations:** 1 Michigan State University, East Lansing, MI, USA; 2 Iowa State University, Ames, IA, USA; 3 National Institute for Media and the Family, Minneapolis, MN, USA

## Abstract

**Background:**

Although several previous projects have attempted to address the issue of child obesity through school-based interventions, the overall effectiveness of school-based programs on health-related outcomes in youth has been poor. Thus, it has been suggested that multi-level interventions that aim to influence healthy lifestyle behaviors at the community, school and family levels may prove more successful in the prevention of childhood obesity.

**Methods/Design:**

This paper describes the rationale, design, and implementation of a community-, school-, and family-based intervention aimed at modifying key behaviors (physical activity, screen time (Internet, television, video games), and nutrition) related to childhood obesity among third through fifth graders in two mid-western cities. The intervention involves a randomized study of 10 schools (5 intervention and 5 control schools). The intervention is being conducted during the duration of the academic year – approximately 9 months – and includes baseline and post-intervention measurements of physical activity, dietary intake, screen time and body composition.

**Discussion:**

We hope this report will be useful to researchers, public health professionals, and school administrators and health professionals (nurses and physical/health educators) seeking to develop similar prevention programs. It is obvious that more collaborative, inter-disciplinary, multi-level work is needed before a proven, effective intervention package to modify behaviors related to childhood obesity can be generally recommended. It is our hope that SWITCH is a step in that direction.

**Trial Registration:**

ClinicalTrials.gov NCT00685555

## Background

The relatively high prevalence and secular increase in pediatric obesity among children and adolescents in the United States over the past few decades are well-known [1]. The convincing evidence for the adverse medical consequences [2], adverse psycho-social consequences [3], and the economic burden [4] of pediatric obesity further identifies the magnitude and significance of this health problem in contemporary society. As a result of the widespread concern of the pediatric obesity epidemic, several key position stands, review papers, and monographs have been published that highlight the importance of effective prevention and treatment programs for pediatric obesity [5-13].

In terms of prevention efforts, the school setting is frequently targeted for intervention programs since it reaches large segments of the youth population [11,14-16]. Guidelines from the Centers for Disease Control and Prevention have specifically emphasized the importance of school programming for modifying physical activity [14] and diet [17] of children. A variety of programmatic changes have been evaluated but the overall effectiveness of school-based programs on health-related outcomes in youth has been poor [18,19]. A main limitation is that many studies have not built in the needed support from families or communities to allow behavior change to be maintained over time. Thus, it has been suggested that multi-level interventions that aim to influence healthy lifestyle behaviors at the community, school and family levels may prove more successful in the prevention of childhood obesity [13,18,20].

A number of social ecological models are available to facilitate planning of multi-level interventions [21-23] but there are few, if any, examples of studies that have implemented a multi-level intervention aimed at addressing the increasing prevalence of overweight and obesity in youth. This paper presents an overview of SWITCH™, a community-, school-, and family-based intervention aimed at changing key behaviors (physical activity, television viewing/screen time, and nutrition) related to childhood obesity. The program was designed and implemented by the National Institute on Media and the Family, Minneapolis, MN. The study design and implementation are explained within this paper.

### Overview of the study design

#### What is SWITCH?

SWITCH is a community-, school-, and family-based intervention aimed at modifying key behaviors (physical activity, television viewing/screen time, and nutrition) related to childhood obesity in third through fifth graders residing in two mid-western cities. The primary objective of the study was to 1) increase the amount of habitual physical activity, 2) reduce the amount of total screen time, and 3) increase the consumption of fruits and vegetables among children enrolled in the intervention. By targeting both physical activity (and inactivity – e.g., screen time) and diet it was hoped that we could create a larger impact on energy balance than could be achieved through efforts to target either component on its own. A secondary objective was to reduce the occurrence of overweight/excessive weight gain during the study period. In addition, the study aimed to increase community awareness about the obesity epidemic and the desired behavior changes. The primary outcome measures used to evaluate the effectiveness of the intervention included pedometer-assessed physical activity, self-reported screen time, and self-reported fruit and vegetable consumption. A number of other measures were obtained to answer questions about effects of media violence exposure, changes in parental monitoring of children's media habits, children's aggressive and pro-social behaviors, and school performance. More information on the assessment of these outcomes is provided in section 6.

There were two project strategies taken to achieve the project goals. The first strategy was to increase community awareness and knowledge about preventing childhood obesity through public education. This campaign was intended to create interest in the schools and communities and to facilitate the implementation of a more targeted intervention strategy. The second strategy was to provide a specific intervention to families of 3^rd ^– 5^th ^graders with school support. The intervention was designed to primarily target families since parents serve as a gatekeeper role in influencing physical activity opportunities and access to food [24]. Additional details on the intervention components are provided in section 5.

The intervention was implemented as a randomized study in 10 schools, 5 of which were assigned to implement the intervention, with the other 5 schools assigned to the control group. The experimental and control schools were matched on socio-economic status and area of the community. The intervention was conducted during the duration of the academic year – approximately 8 months with baseline measurements collected in September 2005 and follow-up measurements in May 2006. An additional measurement period occurred 6-months post-intervention to examine adherence to the program. Measurements were taken according to the timeline in Table 1.

**Table 1:** Measurement timeline for SWITCH.

	**Project Activity**	Sept/Oct 2005	Dec 2005	Feb 2006	Apr/May 2006	Nov 2006
Community	Community survey	X			X	
Child	Child survey	X	X	X	X	X
	Anthropometry	X			X	X
	Physical activity	X	X	X	X	X
Parent	Parent survey	X			X	X
	Parent participation survey		X		X	
Teachers	Teacher survey	X			X	X

SWITCH was organized into four sequential phases. During the first phase of the program, the child with parental involvement established a baseline that identified current health behavior practices and evaluated attitudes and feelings towards making a change in the three key components (Do, View, and Chew). Once families identified their current practices they established long term and short-term goals that fit within their lifestyle.

The second phase of the program focused on making incremental changes reinforced by self- rewards. Each change in behavior towards reaching a self-identified goal was rewarded with goal points or activity points. To receive points children and families engaged in SWITCH activities: making healthy fruit and vegetable recipes, using the activity jar to increase physical activity, and utilizing the screen time box to keep track of time spent in front of screens (television, video games, computer.) The child recorded his or her physical activity (Do), screen time (View), and fruit and vegetable consumption (Chew) on a daily basis. Once the weekly tracker was completed and returned to the classroom, the child was rewarded through the use of incentives.

The participating school teachers at the experimental schools were provided with materials and ideas/examples on ways to include the core concepts for the three key health behaviors across existing curriculum; thus, providing opportunities for teachers to engage students in more active learning. For example, students could graph pedometer steps in math, evaluate caloric intake in science, and create a "Top 10" list of things to do besides screen time in language arts. Supplemental worksheet activities also gave teachers tools to reinforce these concepts during the week. However, it is important to understand that 1) although teachers were provided with the opportunity to integrate some of the ideas into their classroom, it was up to them to choose whether to do so; and 2) SWITCH is not a school-based curriculum.

The third phase of the program was designed to make it easier for families to plan meals, include healthy snacks, and include healthy fruits and vegetables in shopping by providing shopping and mealtime planners. As families made changes they became more health conscious and eager to make further changes. SWITCH provided families with the motivation and tools to make that process easier.

The fourth phase of the SWITCH program focused on maintenance of the health behaviors families established over the eight-month period. New habits of Do, View, and Chew were celebrated and reinforced at an end of project gathering.

### Theoretical model

**Figure 1:**
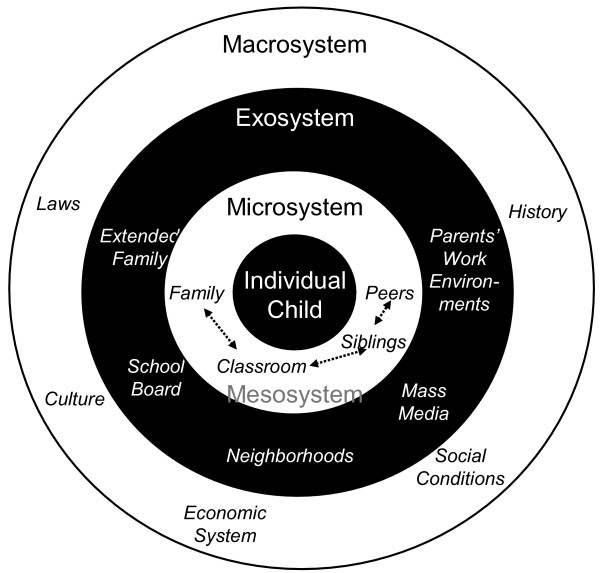
Bronfenbrenner's Ecological Model describing the set of nested environmental influences on a child.

Social ecological models of health promotion have been increasingly used to study the complex interactions affecting individual lifestyle behaviors [21-23]. The social ecological model developed at the Summit on Promoting Healthy Eating and Active Living in 2000 provides a particularly useful framework for examining the complex forces that influence diet and physical activity [21,25] and was used as a guide in the SWITCH project. The model includes a series of concentric rings that describes the various societal and environment factors that influence eating and physical activity patterns as a set of nested environments (see Figure 1). The "*psychobiologic core*" of the model incorporates the genetic, physiologic, and socio-cultural forces that shape our identity. This core is surrounded by cultural and social determinants that directly influence a person's lifestyle behaviors. "*Behavior settings*" are described in the model as physical and social settings in which physical activity and eating behaviors take place or the situational context within which behavior takes place. An established tenet in social-ecological models is that an individual's eating and physical activity behavior is determined by the interactions between the inner social/personal layers and the moderating environmental influences. Because youth spend the majority of their time at home and at school these settings were targeted in the intervention.

The model also proposes that there are multiple leverage points that may be important in modifying nutrition and physical activity behavior [21]. "*Proximal Leverage Points*" refer to the controllers of the structure and features of the micro-environment that affect physical activity and eating behavior choices [21]. In the SWITCH project, the proximal leverage points of interest are family influences. Parents can influence children's eating behaviors by altering types of food available in the home or in restaurants and by altering the ways that food is prepared and consumed. Parents influence children's physical activity behavior by direct efforts to encourage, facilitate or promote activity and by preventing excessive amounts of inactivity. "*Distal Leverage Points*" refer to factors that can shape attitudes, beliefs and behaviors (directly or indirectly) through proximal leverage points. The coding system developed to prioritize possible intervention targets emphasized the relative importance and changeability of different combinations of proximal leverage points and distal leverage points. Families were listed in the final document as one of the more promising leverage points with homes listed as a key microenvironment to reach youth. In the SWITCH project, families and youth were targeted through both school and community programming.

Brofenbrenner's Ecological Model [26] guided the development of the program implementation. This original and seminal social-ecological framework divides the possible sources of influence into different types of environments each nested within the others (Figure 1). The individual child is surrounded most immediately by various microsystem environments. These are the immediate environments with which a child interacts, his or her parents, siblings, teachers, peers, etc. The mesosystem is the relationships that exist between the various microsystems, such as the relations that parents have with a child's siblings and teachers that can affect the target child. The exosystem includes environments with which the child doesn't usually directly interact, but that can still affect the child. These include decisions made by school boards, the opportunities present at the parents' workplaces, etc. The macrosystem includes the broad societal settings under which the others function. These include shared culture, history, or customs, the system of laws, and the economic system. The mass media are often placed at the exosystem level, although a case could be made that the mass media belong at every level. For example, when the child is watching TV, it is part of the microsystem. The siblings and parents also watch and are influenced by TV, and these influences can affect the target child (mesosystem). The media production happens at a level not usually accessible by the child, but those decisions can still affect the child (exosystem). Finally, the media have a large role in shaping culture (macrosystem).

**Figure 2:**
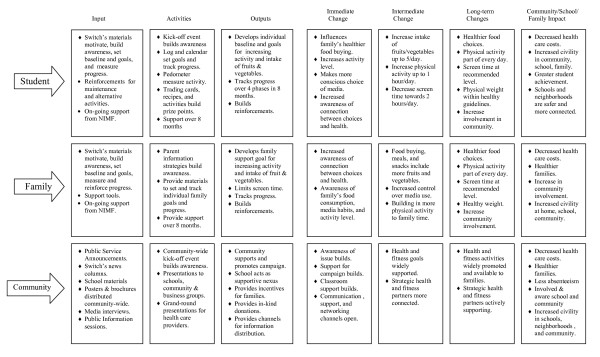
Individual, Family, and Community Change Model Overview for Switch Project.

In designing the SWITCH intervention strategy, we sought to influence youth behaviors and cognitions through three different levels (Community, School and Family). By providing integrated programming at each of these ecological levels, we theorized that we would have a greater impact than a focused emphasis at only one level. Figure 2 provides a schematic view of the intervention strategies and planned outcomes at each of the ecological levels. To conduct this type of multi-level intervention, the intervention team established collaborative efforts with key community members and with the teachers, parents, and school administrators to create an effective program. The objectives of SWITCH therefore target key behaviors related to obesity – physical activity, screen time, and healthful eating. The program promotes a healthy lifestyle by positively influencing 3 types of factors: personal, behavioral, and environmental.

SWITCH aims to enhance individual attributes such as children's knowledge about physical activity and food selections; their values about health, physical activity, and nutrition; and their sense of personal control over their choices. The intervention also seeks to change the behavioral attributes of the children toward a positive choice of exercise and healthful eating, both by adding to their repertoire of activities and lower-fat food items and by teaching them skills that include regular activities and the selection and preparation of foods for themselves and their families. The program also equips children with experience in self-monitoring and goal setting to effect changes in their existing habits; in addition, it offers reinforcement for demonstrating their intention to change, making actual changes, and participating in the program.

Parental obesity is a major risk factor for the development of obesity in children [27]. Furthermore, parents and other family members provide the primary social learning environment in which attitudes and behaviors regarding eating, physical activity, and the use of screen media are formed [24]. The family has a powerful influence over the development and maintenance of children's eating, exercise, and entertainment habits. The strong influence of the relationship between the parent or caregiver and child, including modeling of health behaviors, creating an environment conducive to active or sedentary lifestyles, choosing and preparing food, and encouraging and reinforcing eating and physical activity patterns, suggests that parents and caregivers must be involved in interventions designed to increase healthy eating and physical activity in childhood. Epstein and Wing [28] cited 3 reasons for parental and familial involvement in obesity interventions: *1*) because obesity runs in families, it may be unrealistic to intervene with one family member while other family members are modeling and supporting behaviors that may counteract the intervention's effectiveness; *2*) specific parental behaviors that facilitate overeating and inactivity are important in the development of unhealthy behaviors; *3*) to achieve maximal behavior change in children, use of specific behavior-change strategies (such as positive reinforcement) by parents may be warranted.

### Study communities and participants

General characteristics of the study communities and participants are shown in Table 2. The study communities are located in Lakeville, MN, USA and Cedar Rapids, IA, USA. Lakeville, MN is a community of approximately 50,000 and is considered the southern-most suburb of Minneapolis-St. Paul. Cedar Rapids, IA has a population of approximately 125,000 and is located in east-central IA. These two community school districts comprising ten elementary schools (6 in Cedar Rapids and 4 in Lakeville) were chosen to participate in the study. Schools were matched within district based on average school enrollment and percent free/reduced-cost lunch, and randomly assigned to either an experimental (3 in Cedar Rapids and 2 in Lakeville) or control (3 in Cedar Rapids and 2 in Lakeville) condition. A total of 1359 children were enrolled in the study out of 2091 possible, a 65% participation rate. Participation rates were similar between experimental and control schools, with 685 out of 1019 children participating in experimental schools (67% participation) and 674 out of 1072 children participating in control schools (63% participation). To have sufficient power to detect a difference of 1000 steps/day (standard deviation 2500 steps/day) or 2 fruit and vegetable servings per week (standard deviation 5 servings per week) or 5 hours of screen time per week (standard deviation 12 hours per week) between intervention and control group with power 0.80 and alpha 0.05, a minimum of 200 subjects was needed. Parental consent and child assent were obtained prior to data collection. Teachers also provided consent to complete teacher surveys during the study. All aspects of the study protocol were approved by the University of Minnesota Human Subjects Committee.

**Table 2:** Demographic characteristics of the study populations.

	Lakeville, MN			Cedar Rapids, IA		
	Experimental	Control	Total	Experimental	Control	Total

Population	-	-	23,000	-	-	120,000
N	391	385	776	294	289	583
%White	96%	97%	96%	93%	94%	93%
% with >12 yrs education	96%	94%	95%	87%	82%	85%
Income >$36,000	96%	96%	96%	73%	74%	74%
Income >$100,000	48%	43%	45%	22%	14%	18%
% Married	93%	93%	94%	75%	74%	75%

### Intervention components

#### Community component

The *Community Awareness Strategy *consisted of a public education intervention to increase the targeted communities overall awareness and knowledge about preventing childhood obesity. The first phase of the project began in August 2004 to establish a coalition of community leaders to give the project high visibility, and to advocate for and sustain the project through 2005. The leadership group included leaders and project grantors from education, health care, government, business and the faith communities.

Beginning in January 2005 the following activities commenced and continued throughout 2005.

▪ Launch the project with a community wide event with coalition members and organizations.

▪ Launch a public service advertising campaign in local newspapers and media outlets.

▪ Produce/distribute posters to all types of organizations in the participating school district.

▪ Provide printed materials in community and private family practice and pediatric clinics.

▪ Offer public education/training workshops for parents, teachers, health care providers, religious leaders and business leaders in the community at large.

▪ Offer employee presentations to employers in the community.

▪ Add the project web page to granting organizations web sites.

▪ Provide information to local newspapers for monthly columns and features.

▪ Solicit local businesses to provide incentives and supporting events throughout the project timeline.

▪ Switch Days provided students and families with opportunities to engage in community activities centered around the three goals. (i.e. Swimming, scavenger hunt at local grocery store, roller skating)

▪ Provide public education to existing groups of parents, educators, school boards, etc.

Four hundred randomly selected households in each community were surveyed to ascertain their baseline awareness of the key messages that were included in the community awareness strategies. At the end of the project, a second random set of 400 households were surveyed to measure changes in awareness and/or behaviors in response to the project strategies at the community level.

#### School component

The ***School Strategy ***was a targeted intervention for children in grades 3 through 5 (and their parents or families) to modify the three targeted behaviors: increase in habitual physical activity, reduction of screen time, and increase in consumption of fruits and vegetables. The school setting provides a focused population and environment to exchange information, reinforce positive behaviors in both parents and children, and gather data.

By May 2005, the participating elementary school districts agreed to be project participants and community coalition members. Incentive packages for each school and the participating grades were agreed upon. An orientation for teachers and parent volunteers occurred in August 2005. In September, a school-wide kick-off event was held in participating schools as part of the community wide awareness week. Immediately following the kick-off event, 3^rd ^through 5^th ^grade families were sent a letter of information about the study from the school principals and the principal investigator (D. Walsh). The mailing also included a parental consent form, which allowed their child to be included in the measurement protocols throughout the project. Child assent was also obtained prior to data collection. All classroom teachers who completed teacher surveys also provided consent. At all intervention schools an "Introduction to SWITCH" presentation was given at a scheduled meeting.

The classroom served as one channel for reaching the families and maintaining the Switch message. Teachers were also provided with a monthly teacher's packet which included: posters for the classroom; bulletin board ideas; activity/puzzle handouts for children to do during free time in classroom; a copy of the monthly calendar sent to families. Again, although we provided teachers with examples of curricula integration it was up to the teacher to integrate the ideas into their classroom.

#### Family component

The goal of the family-involvement component was to introduce the child and the adult caregivers to the SWITCH intervention and to assist them in creating a supportive environment for healthy behaviors. Familiarizing family members with the key health behaviors may lead to changes in the household environment. The family component is essential in the development of an environment that supports children's efforts to implement changes in their food choices and levels of physical activity and screen time. To accomplish these goals, the family component consisted of monthly packets which included new materials each month for both the child and the parents. Among the materials included were: a printed brochure describing the project and highlighting the timeline; a printed calendar for the month to help motivate and remind parents about their progress on screen time, activity and nutrition goals designed to easily track each goal; a packet of screen time tickets for the child/parent to track screen time; an activity jar with tips for increasing physical activity; a screen time box with tickets to track the amount of screen time; a meal planner which the families could plan meals and make a grocery list; and recipes that primarily focused on increasing fruits and vegetables in creative and enticing ways that interested children were also provided. These monthly program intervention materials were mailed to families and teachers at the demonstration schools beginning in October 2005 and continued through April 2006. We also provided families with *SWITCH *trackers which allowed families to track their Do, View, and Chew goals on a daily basis over the 8 months.

##### SWITCH points: A means of assessing goals

The overall aim of *SWITCH *was to make it easier for parents and children to choose healthy and active lifestyles. Specifically, *SWITCH *provided practical, easy tips that prompted students to:

*SWITCH up their level of physical activity (DO)

*SWITCH over to choosing more fruits and vegetables (CHEW), and

*SWITCH down hours spent in front of screens (VIEW).

**Figure 3:**
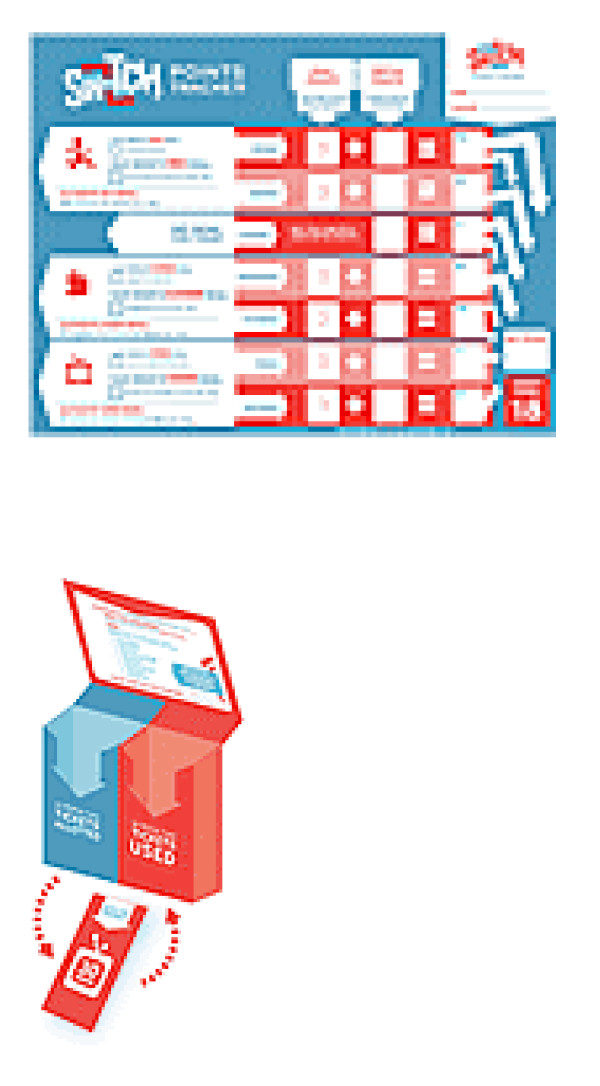
Tracking log and SWITCH box.

The ultimate DO, VIEW, and CHEW goals were to 1) be active for 60 minutes, or more, per day, 2) limit screen time to 2 hours or less per day, and 3) eat 5 fruits/vegetables, or more, per day. The main means for families to assess their progress toward their goals was via SWITCH Points. Students and their families set weekly and monthly goals and received *SWITCH *materials to support their efforts in *SWITCHing *what they DO, VIEW, and CHEW each month. Every activity completed in this project had a SWITCH Point value. The weekly SWITCH Point Tracking Log (see Figure 3) was used to keep track of SWITCH Points. If a student reached the SWITCH Point goal for the week, their name was entered in the SWITCH Point drawing. The child had three choices in setting goals on the SWITCH Point Tracker -increase or maintain physical activity, decrease screen time, and increase or maintain fruit and vegetable consumption.

### Measurement of main outcomes

#### Physical activity

Habitual, free-living physical activity was assessed by a pedometer (Digiwalker 200-SW). The subjects were given instructions on wearing the pedometer during the school day and checked the pedometer using the shaker test and the 10-step test. The shaker test is conducted by simply shaking the pedometer ten times and checking the step count recorded. The 10-step test is conducted by having the subject walk 10 steps and then checking the step count recorded. Pedometers that did not function properly during these 'validity' checks were replaced. Participants recorded the time on/time off and number of steps accumulated over a 7-day period on a customized index card. Participants will be included in statistical analysis only if they have at least 4 days (3 weekday and 1 weekend) when the pedometer was worn for at least 10 hours.

#### Screen time

Children's weekly amount of time viewing television, playing video games, and online computer use was measured with multiple informants: parents and children. Each medium was measured separately, but identically. Parents were asked to indicate the number of hours children watch television between 6 am to noon, noon to 6 pm, and 6 pm to midnight. Because children's viewing habits may be very different on weekdays and weekends during the school year, these three questions were asked separately for weekdays and weekends. These weekday responses are summed and multiplied by 5 (days), and then added to the summed weekend responses multiplied by 2 to create an "average TV viewing hours/week" variable. A parallel set of items were asked for video game use and for online computer use. This measurement approach has been used in other studies [29].

Children were also asked to indicate the number of hours they watch/play on weekdays and weekends for each of the three screen media. However, children's items were anchored by activity rather than by clock time. Children were asked, for example, to report how much time they watch TV between the time they wake up and lunch, between lunch and dinner, and between dinner and when they go to bed. These responses were summed to create average weekly viewing identically to the parent responses. This approach has also been used with children in other studies [30,31].

#### Fruit and vegetable consumption

Children's fruit and vegetable consumption was also measured with two informants: parents and children. Six items assessing the frequency of consumption were adapted from the 2005 National Youth Risk Behavior Survey. The items measured were children's frequency of: drinking 100% juice, drinking soda pop or other sugared drinks, eating fruit, eating green salad, eating carrots, and eating other vegetables. Parents were asked to report for their children with reference to the past seven days. Children were asked to report on their frequency of consumption of each *yesterday*.

#### Anthropometry

Standing height, body mass, and waist circumference were measured by a school nurse according to standard procedures [32]. Standing height was measured using a portable stadiometer (Seca Road Rod). Body mass was measured using a strain gauge scale (Lifesource MD). The body mass index (BMI, kg/m^2^) was calculated from measurements of standing height and body mass. Overweight and obesity were determined based on age- and sex-specific reference values developed by the International Obesity Task Force [33] which are anchored to adult values for overweight and obesity at the age of 18 yrs and back-extrapolated. Waist circumference (WC) was measured above the superior border of the iliac crest as an indicator of central adiposity using a Gullick tape to the nearest 0.1 cm. Prior to data collection, the nurses were trained by the PI and intra- and inter-observer measurement error was determined. In addition, measurement error was also determined during data collection by duplicate measures of every 25^th ^subject. Overall, measurement error was small (SEM = 0.3 cm standing height; 0.1 kg body mass; 0.2 cm WC).

#### Child, parent, and teacher surveys

Because the SWITCH program was designed to work at multiple ecological levels, it was considered critical to gather information from multiple informants at several of those levels. Therefore, surveys were conducted with children, their parents, and their teachers. These surveys included measurement of primary outcome variables as described above (i.e., screen time and fruit/vegetable consumption). However, they also measured several variables of secondary importance.

The children's baseline survey comprised 49 items that measured the following: Children's television violence exposure, children's violent video game exposure, parental monitoring and rules regarding children's television and video games, TV and video games in the bedroom, weekly TV time, weekly video game time, weekly online time, children's attitudes about how much time they spend with TV and video games, amount of daily pleasure reading, incidence of physical fights in the prior year, amount of sleep, snacking habits, attitudes about their physical activity levels, fruit and vegetable consumption yesterday, and self-report of their average school grades. Many of these items were adapted from the General Media Habits Questionnaire and the Adult Involvement in Media questionnaire [30,31].

The parents' baseline survey comprised 64 items that measured the following: The target child's media habits (e.g., frequency of having TV on while doing homework), family media habits (e.g., frequency of having TV on during meals), child and family activities (e.g., frequency of playing games together), parental monitoring and rules regarding children's television and video games, parental consistency with rules for children's media use, TV and video games in the bedroom, children's weekly TV time, weekly video game time, weekly online time, attitudes about how much time the target child spends with media and in physical activity, amount of sleep, snacking habits, the target child's fruit and vegetable consumption over the past week, the child's average school grades, and family demographic variables. Again, many of these items were adapted from the General Media Habits Questionnaire and the Adult Involvement in Media questionnaire [30,31].

The teachers' baseline survey comprised 28 items. Teachers completed one survey for each participating child in their classroom, measuring the following secondary variables: the target child's incidence of physical aggression, relational aggression, and prosocial behavior toward peers, the child's frequency of physical victimization, relational victimization, and prosocial support from peers, the child's attention problems in school, the child's average school grade, and some demographic variables. The aggression and prosocial items were adapted from the Teacher Ratings of Aggressive and Prosocial Behavior Scale [30].

#### Assessment of community awareness

To measure broad community awareness, households with children were randomly selected in the two target communities to participate in a telephone survey (*N *= 400 in each community, total = 800). Parents were asked questions about the relationship between children's screen time, physical activity, and overweight, the relationship between obesity and health issues and, their awareness of the program. Parents were also asked about their children's current media practices, exercise habits, and weight, and about their current behavior regarding their children's media use and exercising. These random surveys were conducted immediately before the SWITCH program implementation, in September 2005, and immediately at the end of the program implementation in June, 2006 (with a new randomly selected sample of 800 parents). The community surveys were conducted by the independent research firm, Anderson, Niebuhr & Associates, Inc.

### Summary

In summary, we have described the background and rationale; study design, measurement procedures, intervention components, and process evaluation procedures. We hope this report will be useful to researchers, public health professionals, and school administrators and health professionals (nurses and physical/health educators) seeking to develop similar prevention programs. It is obvious that more collaborative, inter-disciplinary, multi-level work is needed before a proven, effective intervention package to modify behaviors related to childhood obesity can be generally recommended. It is our hope that SWITCH is a step in that direction.

## Competing interests

The authors declare that they have no competing interests.

## Authors' contributions

JCE, DAG, RC, SS, MW, and DW designed the study, established methods and questionnaires, participated in coordination of the study. GJW provided insight into the conceptual framework of the study. All authors participated in the writing of the paper and provided comments on the drafts and approved the final version.

## Pre-publication history

The pre-publication history for this paper can be accessed here:


